# Strong Room-Temperature Ferromagnetism of MoS_2_ Compound Produced by Defect Generation

**DOI:** 10.3390/nano14040334

**Published:** 2024-02-08

**Authors:** Chang-Soo Park, Younghae Kwon, Youjoong Kim, Hak Dong Cho, Heetae Kim, Woochul Yang, Deuk Young Kim

**Affiliations:** 1 Quantum-Functional Semiconductor Research Center, Dongguk University, Seoul 04620, Republic of Korea; cspark@dongguk.edu (C.-S.P.); hakcho1@dongguk.edu (H.D.C.); 2 Division of Physics and Semiconductor Science, Dongguk University, Seoul 04620, Republic of Korea; uzkym@dongguk.edu (Y.K.); wyang@dongguk.edu (W.Y.); 3 Institute for Rare Isotope Science, Institute for Basic Science, Daejeon 34000, Republic of Korea; kimht7@ibs.re.kr

**Keywords:** MoS_2_, ferromagnetism, Raman, defect, vacancy

## Abstract

Ferromagnetic materials have been attracting great interest in the last two decades due to their application in spintronics devices. One of the hot research areas in magnetism is currently the two-dimensional materials, transition metal dichalcogenides (TMDCs), which have unique physical properties. The origins and mechanisms of transition metal dichalcogenides (TMDCs), especially the correlation between magnetism and defects, have been studied recently. We investigate the changes in magnetic properties with a variation in annealing temperature for the nanoscale compound MoS_2_. The pristine MoS_2_ exhibits diamagnetic properties from low-to-room temperature. However, MoS_2_ compounds annealed at different temperatures showed that the controllable magnetism and the strongest ferromagnetic results were obtained for the 700 °C-annealed sample. These magnetizations are attributed to the unpaired electrons of vacancy defects that are induced by annealing, which are confirmed using Raman spectroscopy and electron paramagnetic resonance spectroscopy (EPR).

## 1. Introduction

Currently, 2D materials are urgently needed for the nanoscale efficient device fabrication and the material-properties engineering arising from their two-dimensional advantages [1,2,3,4,5,6,7]. Transition metal dichalcogenides (TMDCs), among 2D materials, have been chosen to be suitable candidates for nanoscale semiconductor devices [4,5], and TMDCs have extraordinary magnetic [6,7,8,9,10], mechanical [8], electrical [11,12], and optical [13,14] properties. Furthermore, due to its superb electrical characteristics such as the large switching ratio achieved for a device and strong spin-orbit coupling, MoS_2_ has been considered as a promising material for spintronics applications. Therefore, experimental and theoretical studies have reported that ferromagnetism can be generated in MoS_2_ by doping transition metals into MoS_2_ [15,16,17,18,19,20,21]. This kind of material is called diluted magnetic semiconductors (DMSs). These electronic devices using DMSs can have useful magnetic ordering in semiconductors devices for exploiting spin rather than charge [22,23,24,25,26].

On the other hand, the presence of defects can also generate magnetism in pristine or doped MoS_2_ [6,7,8,9,10]. In addition, the annealing can induce a healing process of structural defects. For example, the relationship between vacancies and annealing was studied in single-walled carbon nanotubes, where the semiconducting properties of the field-effect transistor device were improved [27,28].

In this study, we perform annealing on MoS_2_ compound at various temperatures to investigate the effects of defect structure on magnetism. Herein, we report that the MoS_2_ compounds annealed at different temperatures show ferromagnetism without the doping of any transition metal elements. The pristine MoS_2_ without annealing displays diamagnetic behavior. According to our results, the density of sulfur vacancies in MoS_2_ indicates a strong relationship with ferromagnetism, which acts as the core factor for enhancing ferromagnetism in MoS_2_ through annealing. Therefore, this study can shed light on the mechanism of ferromagnetism induced by defects as well as the 2D-based diluted magnetic semiconductor for spintronics devices.

## 2. Materials and Methods

MoS_2_ compounds were purchased from RND Korea (Gwangmyeong, Republic of Korea), and they have a diameter of 800 nm and a thickness of 100 nm on average. We carried out annealing of MoS_2_ compound in a tube furnace and used an alumina crucible for the employment of MoS_2_ compound at the center of the furnace. Before annealing the sample, we purged the furnace with argon gas for half an hour and then heated it up to the intended target temperature (400, 700, and 900 °C) by 5 °C/min at a gas flow of 50 sccm. And the annealing time of the sample was set to one hour under an argon atmosphere. We assigned the name of the annealed samples as the annealing temperature.

The crystal structure, the phase of the samples, and the presence of impurities were evaluated using X-ray diffractometry (XRD, Ultima IV, Rigaku, The Woodlands, TX, USA, Cu Ka radiation). Raman spectra for the compound-samples are evaluated for the excitation wavelength, 514.5 nm, at room temperature, using a spectrometer (Horiba Jobin-Yvon, Oberursel, Germany, HR800UV). X-ray photoelectron spectroscopy (XPS, Versaprobe II ULVAC-PHI) was used for the evaluation of electronic structure. Additionally, the electron paramagnetic resonance spectroscopy (EPR, CW/Pulse EPR System) was utilized to detect the possible sulfur vacancies. And high-resolution transmission electron microscopy (HR-TEM) was measured for the crystal lattice and electron diffraction (JEM-2100 F, JEOL, Tokyo, Japan).

Finally, for the magnetic evaluation of the samples, a superconducting quantum interference device (SQUID, MPMS, Quantum Design, San Diego, CA, USA) was used, and the temperature-dependent magnetization was also measured. The magnetic measurement was performed for the ferromagnetic hysteresis ranging from −3000 to +3000 Oe. The applied fields for the temperature dependence of magnetization are 3000 Oe (FC).

## 3. Results and Discussion

### 3.1. XRD and Raman Spectra

**Figure 1:**
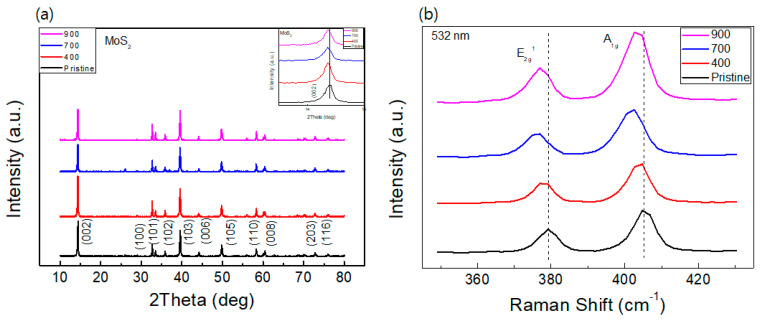
(**a**) XRD diffractograms of the pristine and annealed MoS_2_ compounds, and (**b**) Raman spectra of the pristine and annealed MoS_2_ compounds.

Figure 1a shows the single-phase XRD patterns of pristine MoS_2_ and MoS_2_ compound samples annealed at 400, 700, and 900 °C. The patterns indicate 2H phases (P63/mmc). The redshifts of 0.04 degrees in phases in the (002) peak in the annealed MoS_2_ samples for the pristine MoS_2_ are observed. The enlarged zone of the (002) peak is shown in the inset of Figure 1a for a better comparison. The positions of all the peaks in the three annealed samples are the same, whereas the intensity of the samples is slightly different. This means that the annealing has little influence on the structure of the MoS_2_ compound, and the structural stability is valid for the temperature variation.

Raman spectroscopy has been considered as a powerful measurement technique to explore various properties of two-dimensional materials, such as the transition metal dichalcogenide (TMDC) of MoS_2_. For instance, the doping conditions and the information of layer numbers of 2D flakes can be estimated from the peak shifts or frequency differences of the in-plane mode of E^1^_2g_ and the out-of-plane of A_1g_ modes [29]. As shown in Figure 1b, MoS_2_ compounds have two dominant Raman peaks. The peak of pristine MoS_2_ at ~405.16 cm^−1^ corresponds to the out-of-plane vibration of A_1g_ mode, whereas the peak at ~379.24 cm^−1^ is the result of the in-plane vibration of E^1^_2g_ mode. The thickness of MoS_2_ compound can be identified by measuring the frequency differences between the two modes, the A_1g_ and E^1^_2g_ modes. Our powder samples are approximately 800 nm in diameter and 100 nm in thickness. Here, the frequency difference between the two modes of pristine samples is ~25.92 cm^−1^, which corresponds to the value of bulk MoS_2_ [28]. Some studies also report that the A_1g_ mode of MoS_2_ as well as the E^1^_2g_ mode show a blue or red shift caused by p- or n-type doping [30,31]. Here, two peaks redshift to a lower frequency region as the annealing temperature increases, up to 700 °C, and again shift to a slightly higher frequency region. Although the shape of the peak maxima is not perfect, considering the step for measurement, 2 cm^−1^, the shift is apparent in the low-frequency region. The values of shifts for each spectrum are 2, 4, and 2 cm^−1^ for 400°C-, 700°C-, and 900°C-annealed samples.

This behavior has been shown in previous studies [32]. Sulfur vacancies can be induced owing to sulfur decomposition at highly elevated temperatures, which generate unpaired electrons and n-type doping [33]. Tiny red shifts of 900 °C-annealed sample, restored from the 700 °C-annealed sample, can be seen from the two modes of Raman peaks. Thus, optical healing at a high temperature of 900 °C reduced the density of unpaired electrons, which is consistent with the EPR results, which will be discussed later. However, these shifts are interpreted as the generation of unpaired electrons via annealing.

### 3.2. XPS Spectra

**Figure 2:**
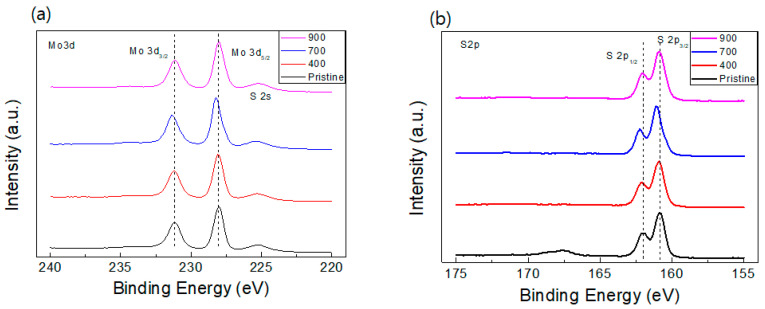
XPS spectra of the pristine and the annealed MoS_2_ compounds for (**a**) Mo 3d and (**b**) S 2p.

XPS is a sensitive and useful tool to identify the electronic structure such as chemical composition and stoichiometry of chemical materials like MoS_2_ compound. Figure 2 shows the core level spectra, demonstrating the binding energy profiles for Mo 3d and S 2p elements of MoS_2_ compound. Three peaks of the XPS spectra, depicted in Figure 2a, recorded at 231.12, 228.01, and 225.09 eV for the pristine MoS_2_ compound are assigned to the Mo 3d_3/2_, Mo 3d_5/2_, and S 2s, respectively. The positions of these peaks indicate a 4+ valence state of Mo from pure MoS_2_ phase, which agrees with another study conducted for MoS_2_ crystals [34]. Compared to the pristine MoS_2_ compound, all annealed compounds indicate no peak shift in binding energy except the 700 °C-annealed sample. It means that the redistribution of the localized charges, owing to the formation of sulfur vacancies, happens only in the 700 °C-annealed sample; sometimes, the introduction of unpaired electrons caused by vacancy generation produces charge redistribution [35], an n-type doping effect, in MoS_2_. However, there is no doping effect in the electronic structure of XPS spectra except the 700 °C-annealed sample. We hypothesize that this may cause a sensitivity problem of XPS for different samples. In Figure 2b, a similar tendency is shown for S 2p_1/2_ and S 2p_3/2_ doublet states, with the binding energy of ~162.02 and ~160.85 eV, respectively.

### 3.3. EPR Spectra and TEM

The increase in vacancy density as annealing temperature increases is identified using the EPR spectra at room temperature together with the Raman shift, which is already mentioned in Figure 1b. The energy difference detected in EPR is attributed to the mutual interaction between the unpaired electron and the external magnetic field. The direction of electron magnetic moment can be parallel or anti-parallel to the magnetic field. Then, the calculation of g-factor can be performed with the following formula; ΔE = gβB_0_ (=hυ). Here, β is the Bohr magnetron, and B_0_ is an external magnetic field.

**Figure 3:**
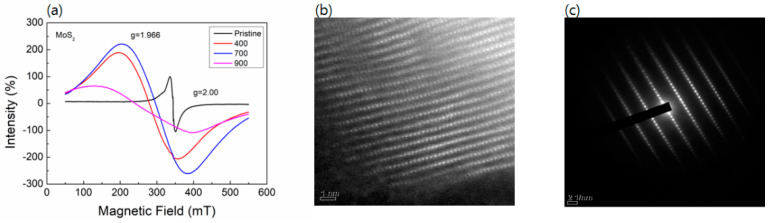
(**a**) EPR spectra of the pristine and annealed MoS_2_ compounds at room temperature and HR-TEM images (**b**) and selected area electron diffraction (**c**) of the 700-annealed sample.

The EPR of the annealed samples displays extremely broad derivative-shaped transitions that are consistent with ferromagnetic resonance. Like the increase in vacancy density in Figure 1b, the paramagnetic signals also demonstrate the intensified signal of unpaired electrons in annealed samples, which is the strongest for the sample annealed at 700 °C. As shown in Figure 3a, the paramagnetic signals around g ~1.966 first increase and then reach their maximum for the 700 °C-annealed sample compound. The EPR data provide further evidence of the sulfur vacancies as the anion vacancies supply the unpaired electrons [36]. The low-EPR intensities of 900 °C-annealed samples indicate that the annealing at 900 °C has negative effects on the productivity of sulfur vacancies in MoS_2_, possibly caused by the structural healing due to high temperatures, which agrees with Raman results.

The TEM measurement was carried out for the estimation of single crystal properties and the existence of vacancy in samples. Figure 3b,c show the TEM image and the SAED pattern of the 700 °C-annealed sample compound. These indicate that the MoS_2_ compound is a single crystal, and some vacancies are shown by dark points, according to lattices in images. Therefore, the observations made based on EPR results are consistent with the TEM data.

### 3.4. Magnetization

**Figure 4:**
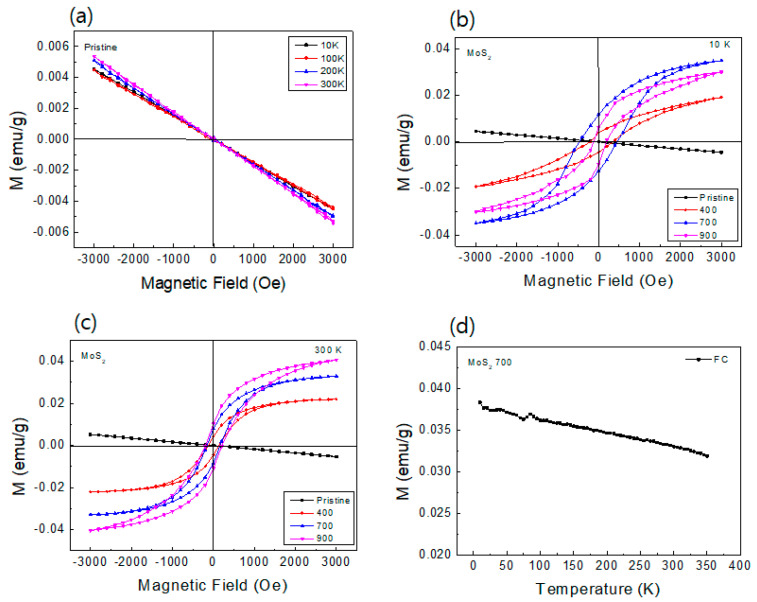
M-H characteristics of the pristine and annealed MoS_2_ compounds from low-to-room temperatures acquired using SQUID. (**a**) Pristine, (**b**) 10 K, (**c**) 300 K, and (**d**) M-T (FC) curve of the 700 °C-annealed sample.

Figure 4 shows the magnetization versus magnetic field (M-H) loops and temperature (M-T) curve of the MoS_2_ compound. As shown in Figure 4a, the pristine MoS_2_ compound indicates diamagnetic behaviors at very low and room temperatures, and this is consistent with the general 2H-MoS_2_ as confirmed by the XRD phase of Figure 1a because the 2H-MoS_2_ has no unpaired electrons. But the all-annealed MoS_2_ compound reveals clear ferromagnetic properties at 10 K and room temperature, as shown in Figure 4b,c. After annealing, the magnetization of MoS_2_ compound gradually increases and exhibits the highest magnetization for the 700 °C-annealed sample at 10 K, as shown in Figure 4b. The ferromagnetic properties of four compounds measured at room temperature are shown in Figure 4c. The 700 °C-annealed sample indicates the highest remnant (M_r_) and saturation magnetization (Ms) of ~0.012 and 0.033 emu/g at 10 K. The 900 °C-annealed MoS_2_ sample seems to possess the second low-temperature Ms, but its tendency is changed in room temperature M-H curves. The magnetization is intensified for the 900 °C-annealed sample, but EPR signals at room temperature, in Figure 3a, show that the density of unpaired electrons is the highest for the 700 °C-annealed sample. Although the magnetization is strongest in the 900 °C-annealed sample at room temperature, we confirm the best magnetization properties are produced in the 700 °C-annealed sample.

According to the theoretical calculations, we know that the ferromagnetism from nanostructure-TMDs is mostly produced by defects, either vacancies or grain boundaries based one-dimensional defects. In some reports of defect-related ferromagnetism in MoS_2_ [37,38], it was suggested that the ferromagnetic properties are attributed to the exchange interactions between the Mo^4+^ ions and sulfur vacancies, and this is called a bound magnetic polaron model. Therefore, according to the results of Raman, SQUID, and EPR, annealing below 900 °C enables an increase in the density of vacancies and leads to higher magnetization. However, further increase in the annealing temperature will heal MoS_2_ structure and decrease ferromagnetic ordering as shown in a previous study [39].

Figure 4d displays the temperature dependence of magnetization of the 700 °C-annealed MoS_2_ compound and represents the tendency of other compounds. The M-T curves were obtained under the applied fields of 3000 Oe (FC). The temperature-dependent magnetization using our MPMS SQUID system has a limitation of increasing temperature. According to the continuous temperature dependence of magnetization above room temperature, the transition temperature, or Curie point, is expected to be higher than 350 K. As a result, the temperature dependence of magnetization (FC) displays a general magnetic compound property at very high temperature [9]. MoS_2_ samples annealed at various temperatures show the tendency of contributing unpaired electrons; higher vacancy density shows higher magnetization with temperature variation.

## 4. Conclusions

In conclusion, we carried out the annealing of MoS_2_ compounds, which can produce ferromagnetic properties due to defect generated by annealing. It is confirmed that the annealing can induce an increase in sulfur vacancies in the MoS_2_ sample, which increases the number of unpaired electrons. The annealed compound shows ferromagnetic properties with Tc above room temperature, in addition to the diamagnetic phases in the pristine sample. The highest remnant and saturation magnetization are achieved in the sample annealed at 700 °C. The 700 °C-annealed MoS_2_ compound has the highest density of vacancies, as confirmed with EPR, suggesting vacancies play a significant role in improved ferromagnetism. This means that the magnetism of TMDCs can be controlled via defect engineering, which provides the valuable routes for the magnetic applications of future spintronic devices.

## Data Availability

Data are contained within the article.

## References

[1] Ahmed S., Yi J. (2017). Two-dimensional transition metal dichalcogenides and their charge Carrier mobilities in field-effect transistors. Nano-Micro Lett..

[2] Nihan K.P., Mehmet B. (2015). Investigation of single-wall MoS_2_ monolayer flakes grown by chemical vapor deposition. Nano-Micro Lett..

[3] Wang Y., Tseng L.-T., Murmu P.P., Bao N., Kennedy J., Ionesc M., Ding J., Suzuki K., Li S., Yiet J. (2017). Defects engineering induced room temperature ferromagnetism in transition metal doped MoS_2_. Mater. Des..

[4] Wu W., Wang L., Li Y., Zhang F., Lin L., Niu S., Chenet D., Zhang X., Hao Y., Heinz T.F. (2014). Piezoelectricity of single-atomic-layer MoS_2_ for energy conversion and piezotronics. Nature.

[5] Wang Q.H., Kalantar-Zadeh K., Kis A., Coleman J.N., Strano M.S. (2012). Electronics and optoelectronics of two-dimensional transition metal dichalcogenides. Nat. Nanotechnol..

[6] Tongay S., Varnoosfaderani S.S., Appleton B.R., Wu J., Hebard A.F. (2012). Magnetic properties of MoS_2_: Existence of ferromagnetism. Appl. Phys. Lett..

[7] Li Y., Zhou Z., Zhang S., Chenet Z. (2008). MoS_2_ nanoribbons: High stability and unusual electronic and magnetic properties. J. Am. Chem. Soc..

[8] Ataca C., Şahin H., Aktürk E., Ciraci S. (2011). Mechanical and electronic properties of MoS_2_ nanoribbons and their defects. J. Phys. Chem. C.

[9] Ahmed S., Viboon P., Ding X., Bao N.N., Du Y.H., Herng T.S., Ding J., Yi J.B. (2018). Annealing effect on the ferromagnetism of MoS_2_ nanoparticle. J. Alloy. Compd..

[10] Shidpour R., Manteghian M. (2010). A density functional study of strong local magnetism creation on MoS_2_ nanoribbon by sulfur vacancy. Nanoscale.

[11] El-Mahalawy S.H., Evans B.L. (1977). Temperature dependence of the electrical conductivity and hall coefficient in 2H-MoS_2_, MoSe_2_, WSe_2_, and MoTe_2_. Phys. Status Solidi B.

[12] El Beqqali O., Zorkani I., Rogemond F., Chermette H., Chaabane R.B., Gamoudi M., Guillaud G. (1997). Electrical properties of molybdenum disulfide MoS_2_. Experimental study and density functional calculation results. Synth. Met..

[13] Wilcoxon J.P., Newcomer P.P., Samara G.A. (1997). Synthesis and otical properties of MoS_2_ and isomorphous nanoclusters in the quantum confinement regime. J. Appl. Phys..

[14] Eda G., Yamaguchi H., Voiry D., Fujita T., Chen M., Chhowallaet M. (2011). Photoluminescence from chemically exfoliated MoS_2_. Nano Lett..

[15] Ganatra R., Zhang Q. (2014). Few-Layer MoS_2_: A promising layered semiconductor. ACS Nano.

[16] Li X., Zhu H. (2015). Two-dimensional MoS_2_: Properties, preparation, and applications. J. Mater..

[17] Andriotis A.N., Menon M. (2014). Tunable magnetic properties of transition metal doped MoS_2_. Phys. Rev. B.

[18] Fan X.-L., An Y.-R., Guo W.-J. (2016). Ferromagnetism in transitional metal-doped MoS_2_ monolayer. Nanoscale Res. Lett..

[19] Zhou J., Li H., Zhang L., Cheng J., Zhao H., Chu W., Yang J., Luo Y., Wu Z. (2011). Tuning magnetism in transition-metal-doped 3C silicon carbide polytype. J. Phys. Chem. C.

[20] Xiang Z.C., Zhang Z., Xu X.J., Zhang Q., Wang Q.B., Yuan C. (2015). Room-temperature ferromagnetism in Co doped MoS_2_ sheets. Phys. Chem. Chem. Phys..

[21] Wang Y., Li S., Yi J. (2016). Electronic and magnetic properties of Co doped MoS_2_ monolayer. Sci. Rep..

[22] Prinz G.A. (1998). Magnetoelectronics. Science.

[23] Ohno H. (1998). Making nonmagnetic semiconductors ferromagnetic. Science.

[24] Ohno H., Shen A., Matsukura F., Oiwa A., Endo A., Katsumoto S., Iye Y. (1996). (Ga, Mn) As: A new diluted magnetic semiconductor based on GaAs. Appl. Phys. Lett..

[25] Shon Y., Kwon Y.H., Yuldashev S.U., Leem J.H., Park C.S., Fu D.J., Kim H.J., Kang T.W., Fan X.J. (2002). Optical and magnetic measurements of *p*-type GaN epilayers implanted with Mn^+^ ions. Appl. Phys. Lett..

[26] Shon Y., Lee S., Yoon I.T., Jeon H.C., Lee D.J., Kang T.W., Song J.D., Yoon C.S., Kim D.Y., Park C.S. (2011). Clarification of enhanced ferromagnetism in Be-codoped InMnP fabricated using Mn/InP:Be bilayers grown by molecular beam epitaxy. Appl. Phys. Lett..

[27] Wang Y., Wang J., Ding C., Zhang H., Du R., Zhang S., Qian J., Hu Y., Huang S. (2020). Laser-induced phenylation reaction to prepare semiconducting single-walled carbon nanotube arrays. Chem. Commun..

[28] Wang Y., Liu D., Zhang H., Wang J., Du R., Li T.-T., Qian J., Hu Y., Huang S. (2020). Methylation-Induced Reversible Metallic-Semiconducting Transition of Single-Walled Carbon Nanotube Arrays for High-Performance Field-Effect Transistors. Nano Lett..

[29] Li H., Zhang Q., Yap C.C.R., Tay B.K., Edwin T.H.T., Olivie A., Baillargeat D. (2012). From bulk to monolayer MoS_2_: Evolution of Raman scattering. Adv. Funct. Mater..

[30] Chakraborty B., Bera A., Muthu D.V.S., Bhowmick S., Waghmare U.V., Sood A.K. (2012). Symmetry-dependent phonon renormalization in monolayer MoS_2_ transistor. Phys. Rev. B.

[31] Mao N.N., Chen Y.F., Liu D.M., Zhang J., Xie L.M. (2013). Solvatochromic effect on the photoluminescence of MoS_2_ monolayers. Small.

[32] Zhao H.-Q., Mao X., Zhou D., Feng S., Shi X., Ma Y., Wei X., Mao Y. (2016). Bandgap modulation of MoS_2_ monolayer by thermal annealing and quick cooling. Nanoscale.

[33] McDonnell S., Addou R., Buie C., Wallace R.M., Hinkle C.L. (2014). Defect-dominated doping and contact resistance in MoS_2_. ACS Nano.

[34] Zhang K., Feng S., Wang J., Azcatl A., Lu N., Addou R., Wang N., Zhou C., Lerach J., Bojan V. (2015). Manganese Doping of Monolayer MoS_2_: The Substrate Is Critical. Nano Lett..

[35] Donarelli M., Bisti F., Perrozzi F., Ottaviano L. (2013). Tunable sulfur desorption in exfoliated MoS_2_ by means of thermal annealing in ultra-high vacuum. Chem. Phys. Lett..

[36] Arizumi T., Mizutani T., Shimakawa K. (1969). EPR study on surface properties of ZnS and CdS. Jpn. J. Appl. Phys..

[37] Cai L., He J., Liu Q., Yao T., Chen L., Yan W., Hu F., Jiang Y., Zhao Y., Hu T. (2015). Vacancy-induced ferromagnetism of MoS_2_ nanosheets. J. Am. Chem. Soc..

[38] Zheng H.L., Yang B.S., Wang D.D., Han R.L., Du X.B., Yan Y. (2014). Tuning magnetism of monolayer MoS_2_ by doping vacancy and applying strain. Appl. Phys. Lett..

[39] Ding X., Liu T., Ahmed S., Bao N., Ding J., Yi J. (2019). Enhanced ferromagnetism in WS_2_ via defect engineering. J. Alloy. Compd..

